# Harmful effect of repetitive intravenous iodinated contrast media administration on the long-term renal function of patients with early gastric cancer

**DOI:** 10.1038/s41598-023-46773-x

**Published:** 2023-11-09

**Authors:** Ja Ho Koo, Myeongjee Lee, Eun Hwa Kim, Hyung Jung Oh, Joon Seok Lim, Woo Jin Hyung, Hong In Yoon, Inkyung Jung, Yong Eun Chung

**Affiliations:** 1 Department of Radiology, Yonsei University College of Medicine, 50-1 Yonsei-ro, Seodaemun-gu, Seoul, 03722 South Korea; 2 Biostatistics Collaboration Unit, Department of Biomedical Systems Informatics, Yonsei University College of Medicine, Seoul, South Korea; 3 Department of Nephrology, Sheikh Khalifa Specialty Hospital, Ras Al-Khaimah, United Arab Emirates; 4 Department of Surgery, Yonsei University College of Medicine, Seoul, Republic of Korea; 5 Department of Radiation Oncology, Yonsei University College of Medicine, Seoul, South Korea; 6 Division of Biostatistics, Department of Biomedical Systems Informatics, Yonsei University College of Medicine, Seoul, South Korea

**Keywords:** Chronic kidney disease, Medical imaging, Computed tomography

## Abstract

This retrospective study investigated whether repetitive exposure to intravenous iodinated contrast media (ICM) affects long-term renal function in patients who undergo curative surgery for early gastric cancer (EGC) collected from the Korean Health Insurance and Review Assessment (HIRA) database. Patients diagnosed with gastric cancer between January 2010 and December 2013 underwent regular computed tomography (CT) scans to monitor for extragastric recurrence. Patients who already had chronic kidney disease (CKD) before cancer diagnosis or had undergone chemotherapy or repeated surgery were excluded. A nested case–control study design was chosen to analyze the effect of repetitive ICM exposure to long-term renal function by comparing patients who developed CKD 2 years after cancer diagnosis and patients who did not. Among 59,971 patients collected according to inclusion and exclusion criteria, 1021 were diagnosed with CKD 2 years after cancer diagnosis. Using 1:5 matching after adjusting for age, sex and date of cancer diagnosis, 5097 control patients were matched to 1021 CKD patients. Conditional logistic regression showed that the number of CTs taken using ICM slightly increased the odds of CKD (odds ratio, 1.080; 95% confidence interval (CI): 1.059, 1.100; P < 0.0001). Thus, the administration of ICM might contribute to chronic renal function impairment.

## Introduction

Computed tomography (CT) is widely used to diagnose cancer and intravenous iodinated contrast media (ICM) is essential for the detection and diagnosis of residual or recurrent gastric cancer or distant metastases^1^. Despite the consensus on its necessity for diagnosis, there is ongoing debate regarding the effects of contrast media exposure on renal function. Some studies have argued that the negative impact of contrast media on renal function may be overstated, even in cases of repetitive exposure^2–4^. However, in other studies, post-contrast acute kidney injury has been associated with negative short-term and long-term outcomes such as longer hospitalizations, higher chances of cardiac and neurologic incidents and higher mortality^5–7^. Although there are still questions regarding the actual risk of iodinated contrast media^8^, it is now evident in animal and human studies that contrast media can contribute to acute kidney injury^7,9–11^. The exact mechanism of nephrotoxicity from iodinated contrast media is not yet fully elucidated, but suggested causes are direct renal damage to the tubular epithelium, high osmolarity and viscosity which trigger increased oxidative stress, vasoconstriction, decreased urine flow and renal contrast media retention, and these factors can finally cumulate in medullary hypoxia and a decreased glomerular filtration rate^12^.

Stomach cancer was reported as the 5th most common cancer and the 4th most common cause of cancer-related deaths in 2020^13,14^. With the development of treatment methods and implementation of a nationwide screening program in South Korea, the overall 5-year survival rates have increased to approximately 75%^15^. Furthermore, the 5-year overall survival rate of early gastric cancer (EGC) has actually been reported as being more than 97%^16,17^. Still, a protocol for CT surveillance after curative gastric cancer surgery has not yet been firmly established. In contrast to colorectal cancer for which intensive follow-up after curative surgery definitely improves overall survival^18,19^, the benefits of intensive follow-up are disputed in patients who undergo curative resection due to gastric cancer^20,21^. Hence, surveillance strategy after gastrectomy varies according to clinician and institution based on individual country guidelines^22,23^.

Recently, concerns have been raised about radiation exposure from CT follow-up scans in patients who undergo curative surgery due to EGC because the extragastric recurrence rate which is the main target of CT surveillance is only 1.4%^24,25^. Most CT scans are performed with contrast administration; hence, the nephrotoxicity of contrast media has to be considered. Therefore, the potential harm from radiation exposure as well as repeated administration of contrast media should be evaluated. There have been many studies regarding post-contrast acute kidney injury per one contrast-enhanced CT^26–29^, but only a few have focused on the harmful effect of repeated contrast administration on long-term renal function^3,4,30^.

Thus, the purpose of this study was to investigate whether repetitive exposure to iodinated CT contrast affects long-term renal function in patients who undergo curative surgery for EGC through an analysis of patients collected from a nationwide database.

## Materials and methods

This retrospective study was approved by the Institutional Review Board of Severance Hospital (No. 4-2019-0252) with the requirement for informed consent waived because of its retrospective nature. This study abided by ethical guidelines stated in the Declaration of Helsinki.

### Data source

Data were retrieved from the Korean Health Insurance and Review Assessment (HIRA) database (HIRA research data M20200206280). The HIRA database stores detailed information on drug prescriptions and treatment procedures performed based on a fee-for-service payment model and this medical information is available on all citizens who have registered for medical insurance in Korea^31^. Data are coded based on the Korean Classification of Diseases, 7th Revision (KCD-7) along with diagnosis, procedure and billing codes. KCD-7 was designed based on the International Classification of Diseases, 10th Revision (ICD-10) and has added a subdivision for frequent diseases that occur in South Korea.

### Patients

Considering that not only chemotherapeutic agents, but also the cancer itself can contribute to kidney injury^32–35^, EGC patients who underwent curative treatment without recurrence during the follow-up period were included in this study. As these patients had minimal initial tumor burden that was removed after treatment and did not receive neoadjuvant or adjuvant chemotherapy^36^, we expected that any effect the cancer and chemotherapeutic agents would have on renal function would be minimized.

Initially, we searched the HIRA database for patients over 20 years old who were diagnosed with gastric cancer (KCD-7 C16) from 2010 to 2013. Among them, patients who underwent curative surgical resection (Procedure code Q0251, Q0252, Q0253, Q0254, Q0255, Q0256, Q0257, Q0258, Q0259, Q2530, Q2532, Q2533, Q2534, Q2535, Q2536, Q2537, Q2591, Q2592, Q2594, Q2595, Q2597, Q2598, QA533, QA534, QA535, QA536, QA595, QA597, QA598) or endoscopic submucosal dissection (ESD) (Procedure code Q7652, Q7653, Q2052, QX701, QX704, QZ933) and who were followed for 5 years after surgery or ESD were included. As an indicator of long-term renal impairment, patients who developed chronic kidney disease (CKD, KCD-7 N18) during the follow-up period were reviewed. Then, the following exclusion criteria were applied: (1) patients who were diagnosed with other malignancies within 2 years before gastric cancer diagnosis (washout period), (2) patients who had an underlying renal disease diagnosed (KCD-7 N00–N19, N25–N29, N39) before gastric cancer diagnosis, (3) patients who underwent any kind of chemotherapy in any time frame, (4) patients who underwent repeated gastric surgery/ESD due to recurring tumors, and (5) patients who were diagnosed with CKD within 2 years after cancer diagnosis to minimize the possibility of CKD developing due to other causes such as subclinical kidney disease rather than contrast media.

### Patient and public involvement

No patients or public were involved in setting the research question or the outcome measures, nor in the interpretation of results.

### Data analysis

In order to control for confounding factors, the presence or absence of diseases known to affect renal function including hypertension (KCD-7 I10.9, I15), diabetes (KCD-7 E10-E14), heart failure (KCD-7 I50), coronary artery occlusive disease (KCD-7 I20-25), stroke (KCD-7 I64), hyperlipidemia (KCD-7 E78.5), and chronic hepatitis virus infection (KCD-7 B18) were investigated. Data on prescription drugs that can affect renal function such as non-steroidal anti-inflammatory drugs (NSAIDs), acetaminophen, loop diuretics, angiotensin II receptor blockers (ARBs), angiotensin-converting-enzyme inhibitors (ACEi), and statins were also reviewed. If medications were prescribed within 1 month of cancer diagnosis and used for more than 2 weeks, they were included. The number of CTs taken using iodinated contrast media (Procedure code HA402, HA403, HA406, HA407, HA410, HA411, HA414, HA415, HA461, HA463, HA464, HA465, HA466, HA467, HA468, HA469, HA471, HA473, HA474, HA475, HA476, HA477, HA478, HA479) during the follow-up period was also analyzed. We compared the number of CTs taken using iodinated contrast media between patients who developed CKD 2 years after cancer diagnosis and patients who did not develop CKD to evaluate the long-term effect of repeated contrast media administration on renal function.

### Statistical analysis

To investigate whether repetitive exposure to iodine CT contrast media affects the long-term renal function of patients who had EGC, a nested case–control study design was chosen. We identified case patients who developed CKD 2 years or later after gastric cancer diagnosis and then identified 4 control patients for each case patient among patients who did not have CKD at the time CKD was diagnosed in the case patient. These control patients were matched so that they were of the same sex and age at cancer diagnosis (within 1 year difference) and were diagnosed in the same year and month as the case patient.

After matching, baseline characteristics between the case group and control group were compared using appropriate statistical methods considering data dependency. A conditional logistic regression was performed to compare baseline characteristics between the case group and control group and to evaluate any existing associations between the number of CTs taken and CKD diagnosis after gastric cancer diagnosis (univariate analysis, Model 1 in Table 2). We further adjusted for prescribed drugs and underlying diseases (multivariate analysis, Model 2 in Table 2). All statistical analyses were performed using SAS Enterprise Guide version 9.4 (SAS Institute Inc., Cary, NC, USA). P-values < 0.05 were considered statistically significant.

## Results

### Patients

**Figure 1:**
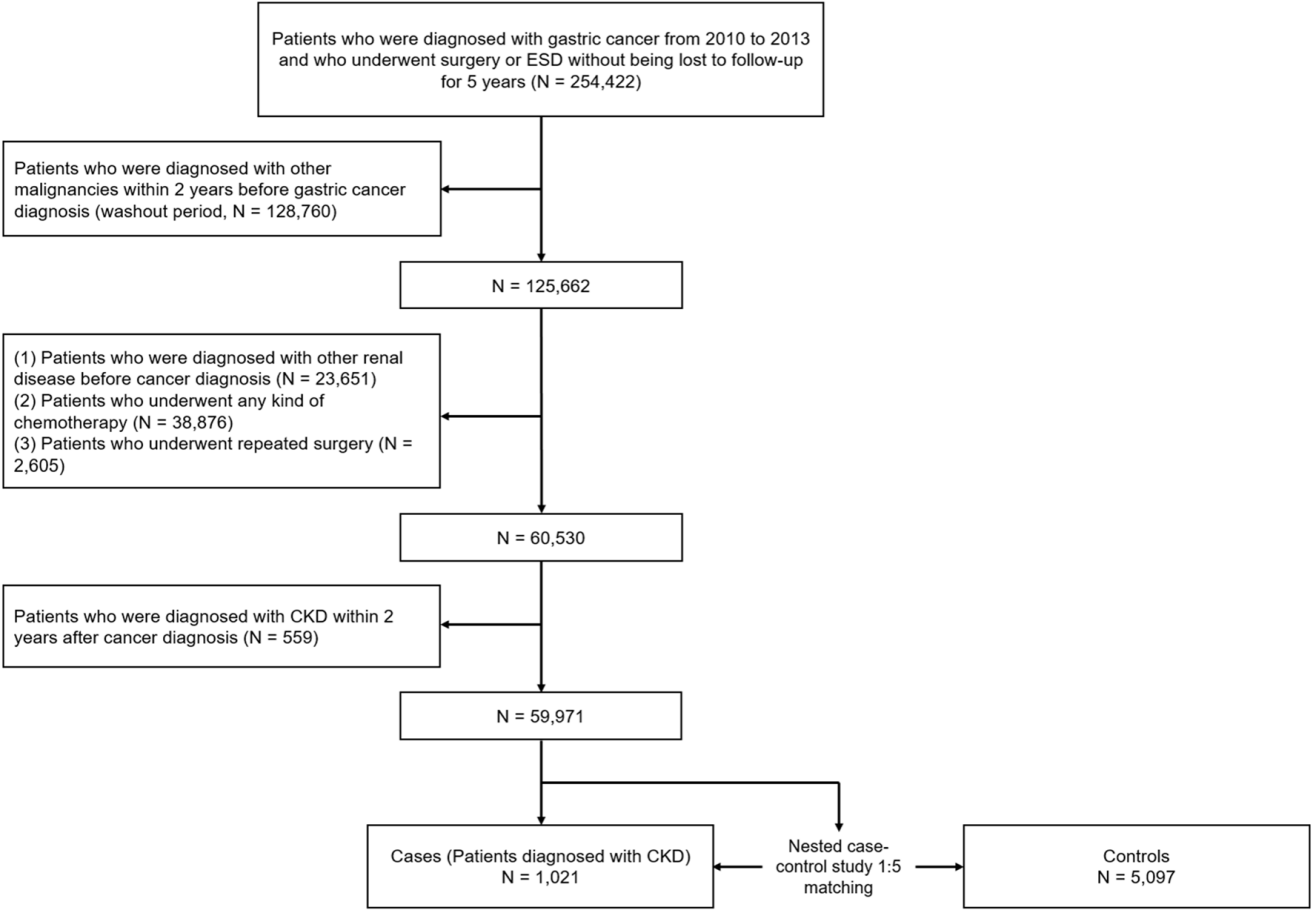
Flow chart depicting patient selection from the HIRA database.

**Figure 2:**
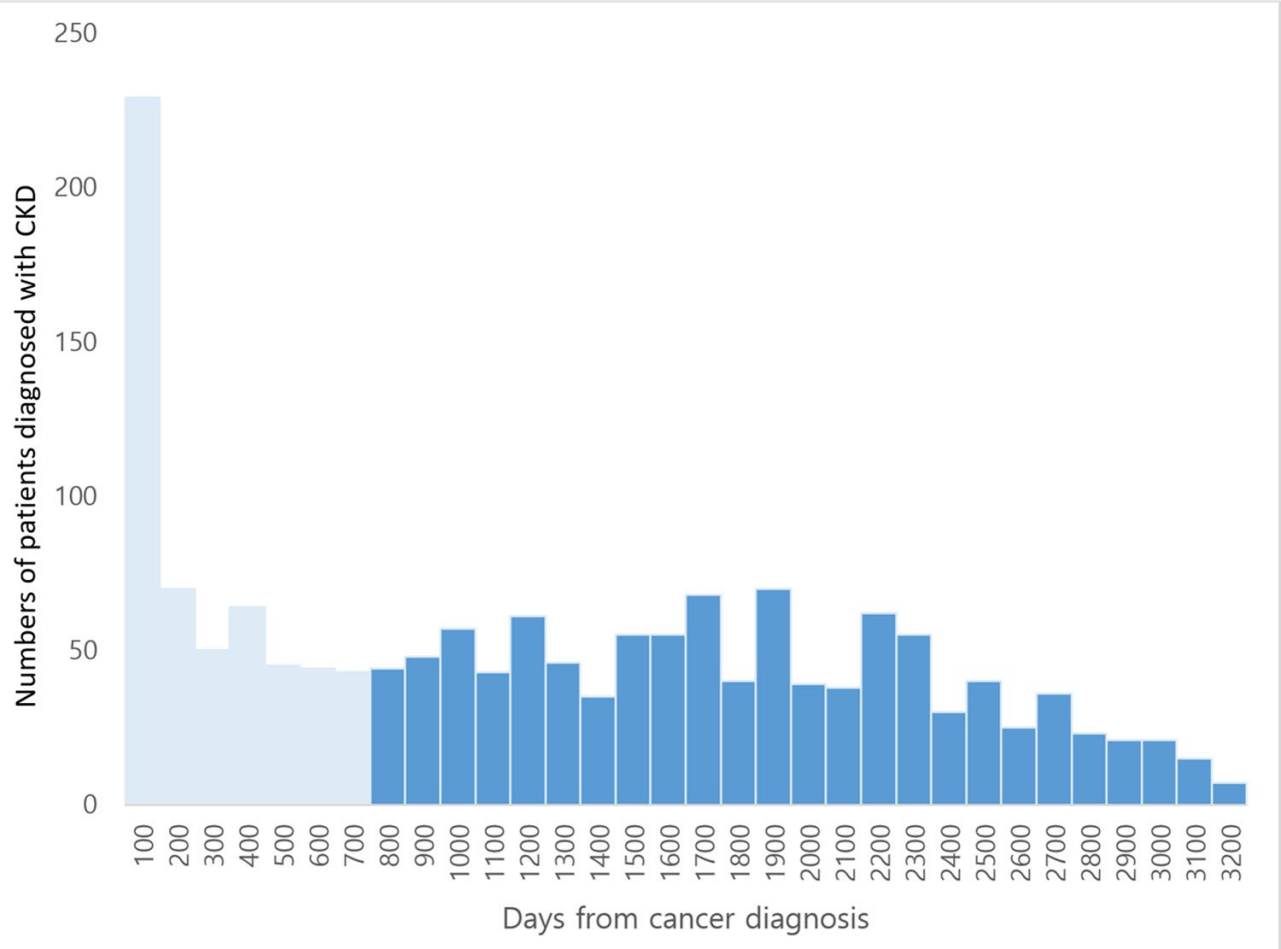
Time interval between CKD diagnosis and gastric cancer diagnosis (light blue indicates patients diagnosed with CKD within 2 years after cancer diagnosis).

There were 254,422 patients who were 20 years old or more and diagnosed with gastric cancer from 2010 to 2013. Among these patients, 128,760 were diagnosed with another cancer during or before the washout period and therefore excluded. Other reasons for exclusion were underlying kidney disease (n = 23,651), chemotherapy (n = 38,876), recurrent surgery or ESD (n = 2605), and a diagnosis of CKD within 2 years after the gastric cancer diagnosis (n = 559). Finally, 59,971 gastric cancer patients were retrieved from the database. Among them, 1021 patients were diagnosed with CKD 2 years after their cancer diagnosis (the CKD group). After 1:5 matching, all CKD patients were matched to at least 1 patient in the control group and 5,097 patients were finally matched to the CKD patients to consist the control group. A detailed flow chart for patient selection is presented in Fig. 1. Baseline characteristics are compared between the CKD and control group after matching in Table 1. Time intervals between CKD diagnosis and EGC diagnosis are presented in Fig. 2.

**Table 1:** Baseline characteristics of patients who were diagnosed with CKD 2 years after cancer diagnosis and matched control patients.

Parameters	Cases (n = 1021)	Controls (n = 5097)	P-value
Age (years)	68.41 ± 9.30	68.40 ± 9.21	0.972
Sex			0.965
Male	795 (77.86%)	3972 (77.93%)	
Female	226 (22.14%)	1125 (22.07%)	
Drugs			
NSAIDs	211 (20.67%)	716 (14.05%)	< 0.001
Acetaminophen	35 (3.43%)	197 (3.87%)	0.249
Loop diuretics	25 (2.45%)	35 (0.69%)	< 0.001
ACEi	27 (2.64%)	94 (1.84%)	0.006
ARBs	131 (12.83%)	339 (6.65%)	< 0.001
Statins	137 (13.42%)	377 (7.40%)	< 0.001
DM	426 (41.72%)	1209 (23.72%)	< 0.001
Hypertension	357 (34.97%)	1226 (24.05%)	< 0.001
Heart failure	49 (4.80%)	119 (2.33%)	< 0.001
CAOD	161 (15.77%)	546 (10.71%)	< 0.001
Stroke	8 (0.78%)	13 (0.26%)	< 0.001
Hyperlipidemia	211 (20.67%)	633 (12.42%)	< 0.001
Chronic hepatitis	30 (2.94%)	115 (2.26%)	0.029
Number of CTs taken	6.02 ± 3.88	4.99 ± 3.73	< 0.001

Case: patients diagnosed with CKD 2 years after cancer diagnosis.

Mean ± standard deviation.

DM: diabetes mellitus; CAOD: coronary artery occlusive disease.

### CT surveillance protocol for gastric cancer

**Figure 3:**
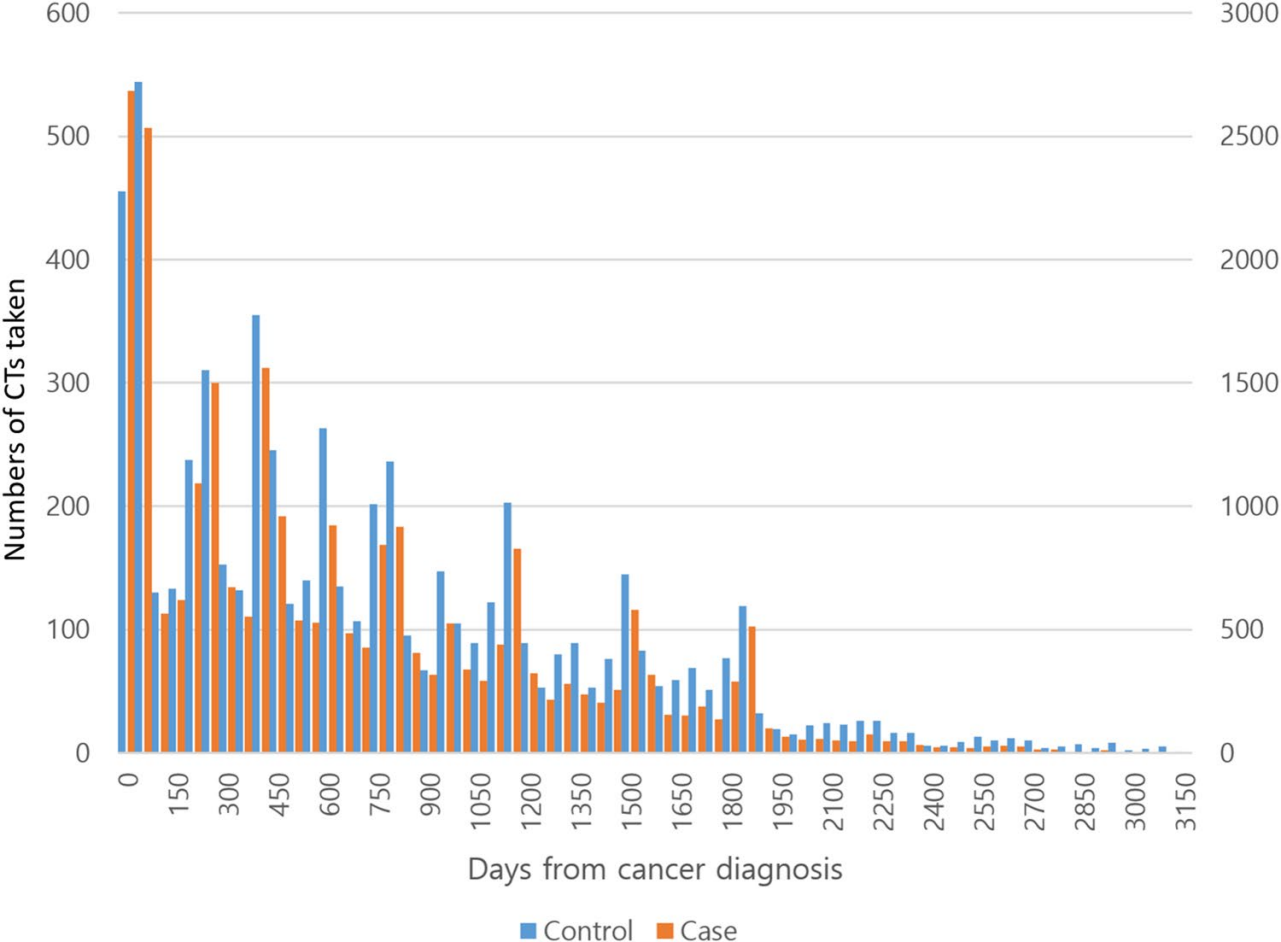
Number of CTs taken from cancer diagnosis according to days from cancer diagnosis in the nested case–control study (the total number of cases was 1021, and the total number of controls was 5097. The left Y-axis indicates the case group, and the right Y-axis indicates the control group) CT scans peaked on the day of cancer diagnosis, and 6 months, 1 year, 1.5 year, 2 years, 3 years, 4 years, and 5 years after cancer diagnosis.

The mean number of CTs taken was significantly higher in the CKD group than the control group (6.02 ± 3.88, 4.99 ± 3.73, respectively, p < 0.001) The number of CTs taken according to days from gastric cancer diagnosis is presented in Fig. 3. As expected, the number of CTs being performed peaked repeatedly 6 months, 1 year, 2 years, 3 years, 4 years and 5 years after diagnosis. The mean number of CTs taken for each patient was 6.12 ± 3.62 (mean ± standard deviation) and the median number was 6 (interquartile range 3–8). The number of CTs taken according to days from gastric cancer diagnosis for all patients before 1:5 matching (n = 59,971) is presented in Supplement Fig. 1.

### Relationship between CKD incidence and the number of CTs taken

Univariate analysis using the conditional logistic regression analysis (Model 1 in Table 2) showed that a higher number of CTs taken with ICM increased the odds of CKD (Odds Ratio (OR) = 1.084; 95% confidence interval (CI) 1.064, 1.104; P < 0.001). The conditional logistic regression analysis for multivariate analysis (Model 2 in Table 2) found that a higher number of CTs taken with ICM slightly increased the odds of CKD (OR = 1.080; 95% CI 1.059, 1.100; P < 0.001) even after adjusting for other confounding factors that are listed in Table 2. Known risk factors for CKD such as hypertension (OR = 1.584; 95% CI 1.312, 1.911; P < 0.001), diabetes mellitus (OR = 1.996; 95% CI 1.714, 2.324; P < 0.001), and hyperlipidemia (OR = 1.349 95% CI 1.109, 1.641; P = 0.003) also increased the odds of CKD developing.

**Table 2:** Associations between the number of CTs taken and CKD.

Model	Parameters	OR (95% C.I.)	P-value
Model 1	Number of CTs taken	1.084 (1.064, 1.104)	< 0.001
Model 2	Number of CTs taken	1.080 (1.059, 1.100)	< 0.001
	NSAIDs	1.135 (0.936, 1.377)	0.199
	Acetaminophen	0.736 (0.503, 1.078)	0.116
	Loop diuretics	2.697 (1.511, 4.815)	0.001
	ACEi	0.913 (0.571, 1.459)	0.704
	ARBs	1.340 (1.057, 1.700)	0.016
	Statins	1.164 (0.915, 1.480)	0.216
	DM	1.996 (1.714, 2.324)	< 0.001
	Hypertension	1.584 (1.312, 1.911)	< 0.001
	Heart failure	1.460 (0.995, 2.143)	0.053
	CAOD	1.012 (0.817, 1.254)	0.914
	Stroke	2.608 (1.051, 6.472)	0.039
	Hyperlipidemia	1.349 (1.109, 1.641)	0.003
	Chronic hepatitis	1.141 (0.743, 1.752)	0.547

Model 1: Conditional logistic regression was used for univariate analysis after matching sex, age at cancer diagnosis and year and month of cancer diagnosis.

Model 2: Multivariate analysis of model 1 with further adjustments for prescribed drugs and underlying diseases. OR: odds ratio; C.I.: confidence intervals.

## Discussion

Our findings indicate that the use of ICM during CT scans may pose a potential risk factor for CKD development, albeit with a relatively low odds ratio of 1.080 for a single CT scan. However, patients diagnosed and treated for malignancies, including EGC, often undergo repeated contrast-enhanced CT scans for a minimum of five years. Thus, the significance of even the modest odds ratio associated with CKD development cannot be understated.

In previous research, iodinated contrast media was considered the cause of post-contrast acute kidney injury, and was linked to negative short-term and long-term outcomes, including prolonged hospitalizations, higher rates of cardiac and neurologic incidents, and increased mortality^5,6,9^. Nevertheless, recent studies have challenged the notion that contrast media significantly impacts renal function, even with repetitive exposure. These studies primarily focused on post-contrast acute kidney injury or the long-term effects of iodinated contrast media over a shorter time frame of 3–6 months^2–4^. In contrast, our study examined the long-term effects of iodinated contrast media over a span of 5 years. As a result, our study demonstrated that exposure to ICM increases the subtle yet statistically significant odds of developing CKD.

Post-contrast acute kidney injury due to iodinate contrast media can lead to the acute deterioration of renal function and this can happen within 48 hours to 7 days after the contrast media administration^37,38^. Traditionally, although acute kidney injury increases both morbidity and mortality, it is considered as a single independent episode and patients who recover from acute kidney injury are thought to have benign long-term outcomes^39,40^. However, recent observational studies have shown that acute kidney injury and CKD are related and that acute kidney injury is an independent risk factor for CKD. Furthermore, the severity, duration and frequency of acute kidney injury are important predictors of poor outcomes^39,40^. If an event of acute kidney injury breaks the balance between adaptive and maladaptive repair mechanisms, acute kidney injury can progress to CKD^39,41,42^. In our study, the odds ratio for CKD development from a single CT scan was relatively small (1.080). Nevertheless, for patients who require repeated CT scans to monitor disease progression or evaluate treatment response, this odds ratio cannot be considered negligible.

The patients in our case group exhibited a notably higher prevalence of comorbid conditions, including hypertension and diabetes mellitus, in comparison to our control group, as detailed in Table 1. Hypertension and diabetes mellitus are widely recognized as risk factors for CKD^43^. Consequently, these baseline characteristics can potentially influence our findings. Therefore, to mitigate the impact of these confounding factors, we conducted a multivariate analysis employing conditional logistic regression. The multivariate analysis demonstrated that both hypertension and diabetes mellitus were associated with an increased odds ratio, in addition to the small but significant impact of ICM exposure. Future studies about the utilization of contrast-enhanced CT scans in patients known to possess risk factors for CKD, such as hypertension and diabetes mellitus are warranted.

Usually, endoscopy is regularly performed to detect mucosal recurrence or metachronous gastric cancer during surveillance^44^. The role of CT in gastric cancer follow-up is to detect extragastric recurrence, but Seo et al. stratified the risk for extragastric recurrence as high and low in EGC patients based on five risk factors which were male sex, lymph node metastasis, positive lymphovascular invasion, indications for endoscopic resection, and elevated macroscopic type and reported that only 0.265% of EGC patients at low risk of extragastric recurrence suffered extragastric recurrence in a 10-year period and that the 10-year extragastric recurrence-free survival was very high at 99.7%. Thus, they concluded that CT surveillance to detect extragastric recurrence should be avoided in the low risk group^24^. For patients with a very low risk of cancer recurrence, there is a need to weigh the diagnostic value of CT against the harmful effect of contrast media exposure to long-term renal function. In our study, there was significant difference of CKD development between case and control groups, albeit, the difference of mean number of CT taken was just approximately one scan (6.02 versus 4.99). Thus, the surveillance period and CT scan interval should be adjusted and redesigned based on future research.

According to a previous study, approximately 44% of elderly adults 65 years or older have CKD, but only 7% have been diagnosed in the United States^45^. These numbers are comparable with our results as 229 patients among 1021 patients (14.5%, Fig. 2) were diagnosed with CKD at the same time gastric cancer was diagnosed, which suggests that these patients already had CKD, but did not undergo relevant examinations until perioperative renal function assessment. Thus, CKD was undetected and overlooked in many cases. The washout period has been widely used in national databases to define new incident cases and eliminate any potential prevalent cases^46^. In a similar manner, we excluded patents who were diagnosed with CKD within 2 years of cancer diagnosis to minimize the possibility of CKD developing from other underlying medical conditions rather than contrast media. In patients who have not yet been diagnosed with CKD but may be experiencing deteriorating renal function due to etiologies other than those related to contrast media, we must consider these patients as confounding variables if CKD is diagnosed later after the administration of contrast media. Given that CKD is a condition that progresses gradually over a long period of time, we established a substantial washout period of two years to mitigate the inclusion of such patients in our study.

Global guidelines usually recommend a CT follow-up every 6 months for 2 years after surgery, then an annual CT follow-up thereafter for 5 years^22,23,47^. This general protocol is reflected in our study with CT follow-up examinations peaking at 6 months, 1 year, 2 years, 3 years, 4 years and 5 years after diagnosis as shown in Fig. 3 and Supplement Fig. 1. In our study, the number of CTs taken was the highest just after diagnosis and we assumed that this was because CT scans were performed to check for postoperative complications in the perioperative period.

There are several limitations to this study. First, the HIRA data did not included specifics on the iodinated contrast media such as the administered volume and osmolality of the contrast media which have also been reported as risk factors for kidney injury^12,48^. Also, eGFR data was not available and CKD was defined with the KCD-7 codes. Furthermore, specific pathologic stages were not recorded in the HIRA database. Second, heterogeneity due to different institutional guidelines and CT protocols needs to be considered when analyzing the HIRA data. Third, a selection bias might exist because physicians try to minimize the use of contrast media in patients who at higher risk of renal injury. Fourth, more severe postoperative complications may lead to more CT scans, which might subsequently have affect renal function. However, the HIRA database referred to in this study does not store detailed information on complications, so the severity of postoperative complications on renal function could not be analyzed in this study. Finally, we could not collect follow-up data that extended past 10 years of the ICM administration. Studies based on data collected from a longer follow-up period are warranted.

In conclusion, the administration of iodinated contrast media may be a potential risk factor for long-term renal impairment. Hence, the risks and benefits of contrast-enhanced CT need to be reevaluated in patients who undergo curative surgery and who are at low risk of recurrence such as those with EGC.

### Supplementary Information

Supplementary Figure 1.

## Data Availability

The data that support the findings of this study are available from HIRA but restrictions apply to the availability of these data, which were used under license for the current study, and so are not publicly available. Data are however available from the authors upon reasonable request and with permission of HIRA.

## Abbreviations

HIRA: Korean Health Insurance and Review Assessment
EGC: Early gastric cancer
CKD: Chronic kidney disease
ICM: Intravenous iodinated contrast media
