# Supplementary material for: Harmful effect of repetitive intravenous iodinated contrast media administration on the long-term renal function of patients with early gastric cancer
Source: Sci Rep. 2023 Nov 9;13:19448. doi: 10.1038/s41598-023-46773-x (PMC10636198; doi:10.1038/s41598-023-46773-x)
Supplement: Supplementary file 1 — Supplementary Figure 1. [file 41598_2023_46773_MOESM1_ESM.docx]

Supplement Figure 1


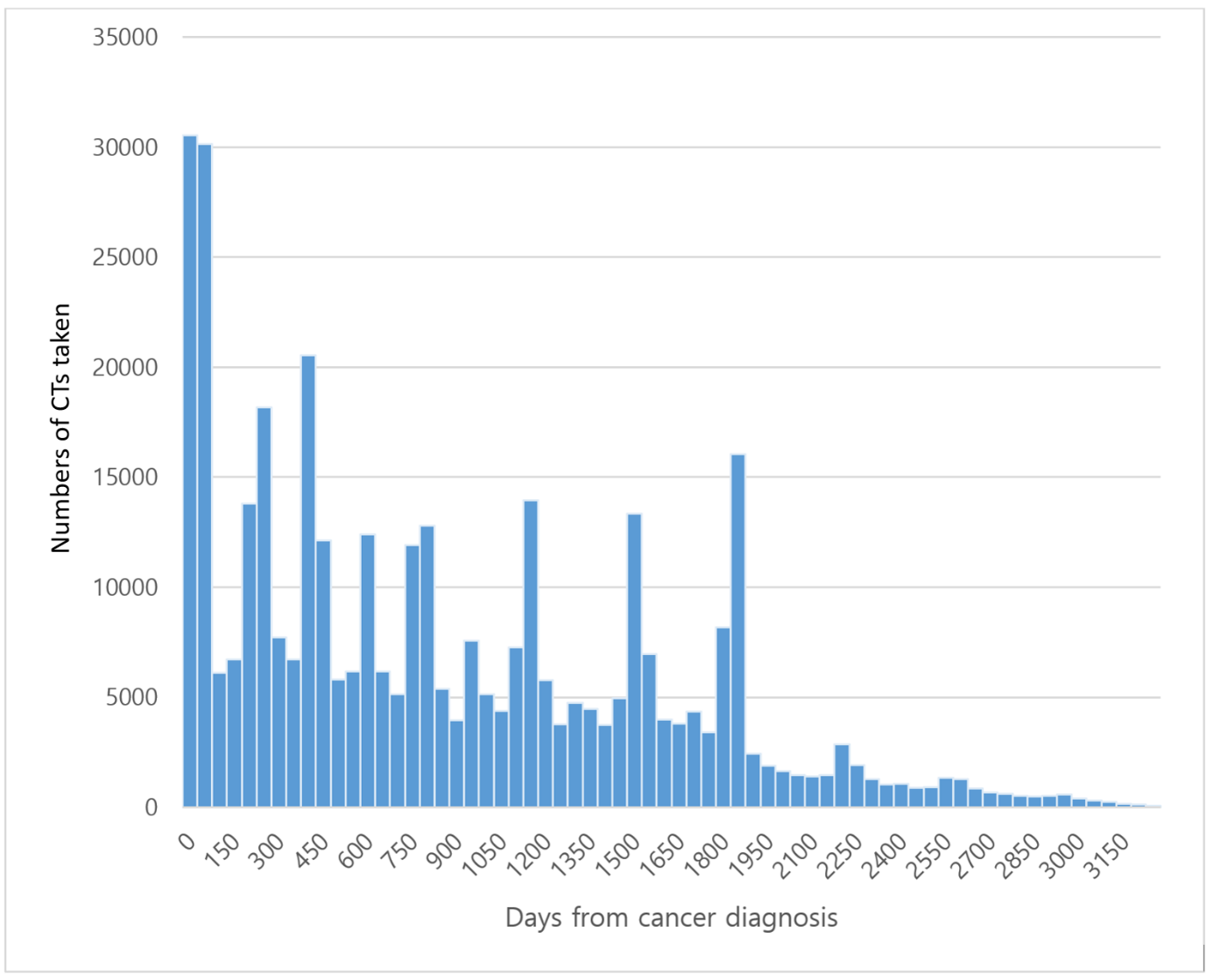


**Supplement Figure 1. The number of CTs taken from cancer diagnosis according to days from cancer diagnosis in all patients before the nested case-control study.**
